# Roles of viable but non-culturable state in the survival of *Campylobacter jejuni*

**DOI:** 10.3389/fcimb.2023.1122450

**Published:** 2023-03-28

**Authors:** Leticia Silva Santos, Daise Aparecida Rossi, Raquelline Figueiredo Braz, Belchiolina Beatriz Fonseca, Micaela Guidotti–Takeuchi, Rosiane Nascimento Alves, Marcelo Emílio Beletti, Hebreia Oliveira Almeida-Souza, Larissa Prado Maia, Paula de Souza Santos, Jéssica Brito de Souza, Roberta Torres de Melo

**Affiliations:** ^1^ Laboratory of Molecular Epidemiology, Federal University of Uberlandia, Uberlandia, Brazil; ^2^ Biotechnology Institute, Federal University of Uberlandia, Uberlandia, Brazil; ^3^ Institute of Biomedical Sciences, Federal University of Uberlandia, Uberlandia, Brazil

**Keywords:** adaptation, campylobacteriosis, latency, metabolomics, SEM, TEM, virulence

## Abstract

Despite being considered fragile and fastidious, *Campylobacter jejuni* is the most prevalent cause of foodborne bacterial gastroenteritis, and chicken meat is considered the main vehicle of transmission to humans. This agent can survive adverse conditions in the form of biofilms, but extreme stress (nutritional, oxidative and thermal) promotes the acquisition of a state called viable but not culturable (VBNC). The emergence of this pathogen worldwide and the recent international requirements in its control instigated us to qualitatively and quantitatively estimate the time required for the acquisition of the VBNC form in 27 strains of *C. jejuni*, characterize morphological aspects, determine its adaptive and invasive potential and perform comparative metabolomic evaluation. Extreme stress promoted the complete acquisition of the VBNC form in a mean time of 26 days. Starting from an average initial count of 7.8 log CFU/mL, the first four days determined the greatest average reduction of the culturable form of 3.2 log CFU/mL. The scanning and transmission image analyses showed a transition from the typical viable form (VT) to the VBNC form, with initial acquisition of the straight rod shape, followed by loss of the flagella and subdivision into two to 11 imperfect cocci arranged in a chain and rich in cellular content, until their individual release. RT-PCR identified the presence of *ciaB* and *p19* transcripts in the 27 cultivable *C. jejuni* strains, a character maintained in the VBNC form only for *p19* and in 59.3% (16/27) of the VBNC strains for the *ciaB* gene. The average inoculation of 1.8 log CFU/mL of *C. jejuni* VBNC into primary chicken embryo hepatocyte cells promoted the occurrence of apoptosis processes significantly after 24 hours of contact by one of the strains tested. In *C. jejuni* VBNC, we detected higher expression of metabolites linked to protective and adaptation mechanisms and of volatile organic precursor compounds indicative of metabolism interruption. The oscillations in the time of acquisition of the VBNC form together with the presence of transcripts for *ciaB* and *p19*, the identification of cell lysis and metabolites that ensure the maintenance of the pathogen alert to the fact that *C. jejuni* VBNC remains virulent and adapted to stress, which makes evident the potential danger of this latent form, which is not detectable by official methodologies.

## 1 Introduction

*Campylobacter jejuni* belongs to the Campylobacteraceae family and is the most prevalent agent in foodborne gastroenteritis in Europe and the US, associated not only with enteric conditions, but also with autoimmune changes such as Guillain-Barré Syndrome. Among the routes of human infection, chicken meat represents the main source (EFSA, 2016; CDC, 2017), and in Brazil, poultry farming stands out for the title of largest exporter and third largest producer (ABPA, 2022). But, due to the difficulties in diagnosing campylobacteriosis and underreporting problems in the country, the few records of the disease in humans are related to occasional research (Cisco et al., 2017).

Despite the progress related to the inclusion of regulations and legislation for the control of this microorganism in chicken meat, the detection methodologies used are still based on the identification and quantification of the pathogen by traditional microbiological tests (ISO, 2017). This is particularly important because *Campylobacter* can enter a viable but non-culturable (VBNC) state after exposure to various stresses, including low temperature, presence of oxygen, salt treatment and reduced nutritional intake (Silva et al., 2011), situations common in foods available to consumers. VBNC cells are unable to multiply in conventional culture medium when there are changes in membrane integrity to a coccoid shape, reduced metabolic activity and subsequent latency stage (Ramamurthy et al., 2014).

Furthermore, it has been shown that *Campylobacter* can exhibit complex resistance and survival advantages such as stress response mechanisms that help maintain virulence, osmoregulation, protection against oxidative stress, thermotolerance, and heat shock response (van Vliet and Ketley, 2001; Pascoe et al., 2015) that assist in maintaining strains in natural environments, extending transmissibility, and the ability to colonize multiple hosts (Sheppard et al., 2009)

In addition, responses against adverse conditions (oxygen exposure, starvation, and osmolarity fluctuations) (Garénaux et al., 2008; Yahara et al., 2017) induce biofilm formation (Pascoe et al., 2015) and the determination of morphological changes such as the transition to VBNC (Bronowski et al., 2014). The largely culture-dependent methods severely underestimate the presence of VBNC cells. Under this condition, the bacterium can remain inactive for indeterminate periods and reassume the infecting form under favourable conditions. VBNC cells are inferred to be avirulent due to a reduced rate of gene expression, yet VBNC cells that are resurrected can regain full infectious phenotypes (Baffone et al., 2006; Pinto et al., 2015), posing a real threat to public health (Lv et al., 2020).

Given the evidence, we were instigated to qualitatively and quantitatively estimate the time required for the complete acquisition of the VBNC form in 27 C*. jejuni* strains, characterise morphological aspects, determine their adaptive and invasive potential and perform comparative metabolomic evaluation, considering the possible risk to the consumer.

## 2 Materials and methods

### 2.1 Strains and conditions

Twenty-seven phylogenetically distinct *C. jejuni* strains, cryopreserved at -80°C and from chicken carcasses, isolated by Melo et al. (2019c) were reactivated on CCDA agar (Oxoid^®^). The strains evaluated were previously explored by phenotypic antimicrobial resistance, virulence profile and RAPD-PCR and were considered different strains in a previous study (Rossi et al., 2021). They were collected from chicken carcasses from the Brazilian poultry industry, isolated between the years 2015 to 2016, three states in two different regions in the country. Cells from the stock culture were reactivated on mCCDA agar (Oxoid) under microaerophilic conditions at 37°C for 48 hours. Then, approximately 20 colonies were transferred from the subculture in mCCDA to 20 mL of Mueller Hinton (MH) broth (Oxoid^®^) and incubated for 48 hours at 37°C in microaerophilic conditions, followed by centrifugation for 5 minutes at 10,000 rpm at 4°C. The pellet was washed three times in 0.9% saline, and the resulting suspension was standardized to an OD_600_ of approximately 0.4 in sterile saline equivalent to about 7–8 log CFU/mL (PGInstruments^®^), as confirmed by plate count and used as the standard inoculum for obtaining the VBNC form. All assays were done in three replicates and three independent replicates, and the standard IAL 2383 strain of *C. jejuni* was used as a control for viability and culturability.

### 2.2 Quantification and obtention of the VBNC form

The strains in saline suspension were incubated at 4°C for up to 60 days in conditions of oxygen presence, simulating thermal, nutritional and oxidative stress to induce the VBNC state, according to Magajna and Schraft (2015).

The method used to evaluate culturability was conventional counting on CCDA agar plates, accompanied by morphological evaluation in light microscopy, performed every four days (0, 4, 8, 12, 16, 20, 24, 28, 32, 36, 40, 44, 48, 52, 56 and 60) in three replicates per strain, in three repetitions. In parallel, BacLight biovolume analysis was used for one of the replicates. Day 0 corresponded to the cells (VT form) before exposure to the stress conditions and was used in the comparison. On each assay day, the tubes with samples to be tested were removed and vortexed briefly, and then representative aliquots of 10 serial dilutions were taken for each test, followed by returning the tubes to 4°C. For assessment of culturability, 1 mL of the dilutions were transferred to mCCDA plates. The plates were incubated micro-aerobically at 37°C for 48 hours for further quantification.

### 2.3 Cell viability monitoring

The LIVE/DEAD BacLight staining assay (Thermo Fischer) was used to obtain total cell counts in one of the replicates for the 27 strains. A 5 mL aliquot of each sample was stained with the fluorescent dyes propidium iodide (PI, 20 mM in dimethyl sulfoxide) and SYTO 9 (3.34 mM in dimethyl sulfoxide) in a 2:1 ratio. Samples were incubated with 15 μL of dye mixture at 37°C in the dark for 15 minutes and then immediately transferred a volume of 200 μL to 96-well microplates in triplicate. Results were read using fluorescence spectrophotometry (Biotec), according to the protocol described by Ou et al. (2019) and using the formula: %live cells = SYTO9/PI, where SYTO 9 and PI represent the integrated intensity of SYTO 9 and PI, respectively. The intensity integration regions corresponded to the fluorescence peak of the dyes, which were between 509–529 nm for SYTO 9 and 609–629 nm for PI. Mathematically, the %live cells are equivalent to the number of live cells divided by the total number of cells, which is not reflected by SYTO 9/PI. The reason for this becomes clear when assuming the ideal behaviour of the dyes where live and dead bacteria will be marked mainly by SYTO 9 and PI, respectively (ThermoFisher Scientific. LIVE/DEAD®, 2004).

### 2.4 Scanning electron microscopy

The preparation of the material for analysis in SEM was done, according to Brown et al. (2014), with modifications. For the evaluation of external morphology, three *C. jejuni* VT and VNBC strains in suspension were randomly selected, subjected to washing and centrifugation at 10,000 g for 5 minutes at 4°C. 100 μL of the pellet was pipetted onto a 1 cm^2^ polyurethane slide coated with a 0.22 μm pore size cellulose membrane (Millipore^®^). Slides were dried at room temperature for 15 minutes and fixed with 2.5% glutaraldehyde and 2.5% paraformaldehyde in 0.1 M PBS buffer (pH 7.4) overnight at 4°C. The fixative was removed, and the samples were washed three times with PBS buffer. Slides were post-fixed with 1% osmium tetroxide for two hours and washed three times with PBS buffer. The slides were then dehydrated in a series of ethanol solutions (30, 40, 50, 60, 70, 80 and 90% and then three times at 100%) for 15 minutes for each step. The samples were dried in a CPD (Critical Drying Point) (CPD 030, Baltec, Germany), using liquid carbon dioxide as the transition fluid. The coating was made with a 20 nm thick layer of gold (SCD 050, Baltec, Germany) and visualized on a VP Zeiss Supra 55 FEG SEM operating at 20 kV.

### 2.5 Transmission electron microscopy

For the evaluation of internal morphology, three *C. jejuni* strains in VT and VNBC forms in suspension were randomly selected. Initially, the samples were fixed in Karnovsky’s solution (2.5% glutaraldehyde; 2% paraformaldehyde; 0.1 M sodium cacodylate buffer at pH 7.2) for 1 hour, centrifuged (2,000 g) to decant the cells for 5 minutes and then the supernatant was discarded. Then, the samples were extensively washed in sodium cacodylate buffer (0.2 M) for 6 hours, centrifuged and included in gelatin (0.5%).

The material was post-fixed in a 1% osmium tetroxide solution for 1 hour and subjected to dehydration in an increasing series of alcohol at 50%, 70%, 80%, 90%, 95%, 100%, 100% and 100% for five minutes in the first five baths and ten minutes in the last three. For the final dehydration, three 10-minute baths in propylene oxide were applied. Subsequently, the material was placed in a solution of Epon resin and propylene oxide in the proportions 2:1 and 1:1 for 12 and 6 hours, respectively. After this period, the solution containing the material was placed in an oven at 37°C for 12–24 hours.

After evaporation of all propylene oxide, the blocks were embedded in pure resin and kept for two days in an oven at 60°C. Finally, ultrathin sections were obtained using the ultramicrotome, which were contrasted with uranyl acetate and lead nitrate and analyzed in the transmission electron microscope (TEM) (HITACHI HT7700, Tokyo, Japan) (Bozzola and Russell, 1998).

### 2.6 Gene transcription analysis

Strains were evaluated for the ability to produce transcripts in typical and VBNC forms. For this purpose, RNA extraction was performed using the Trizol method as described by Li et al. (2008), with modifications. A bacterial suspension was centrifuged at 12,000g for ten minutes at 4°C. To the obtained pellets, 1 mL of Trizol (Invitrogen^®^) was added and homogenised by vortexing (Phoenix^®^). Subsequently, 200 μL of chloroform (Sigma Aldrich^®^) was added and the vortexing homogenisation procedure was repeated, followed by centrifugation at 12,000g for 15 minutes at 4°C. The aqueous portion formed was transferred to a new microtube, to which 500 μL of isopropanol (Sigma Aldrich^®^) was added, homogenised and centrifuged again at 12,000g for 10 minutes at 4°C. To the pellet formed, 1 mL of 75% ethanol (Sigma Aldrich^®^) was added and then homogenised and centrifuged at 7,500 g at 4°C for 5 minutes; the supernatant obtained was discarded. The RNA pellets were dried at room temperature to be diluted in 20 μL of DEPC water (Invitrogen^®^). The RNA concentration used was 200 ng/μL, which was quantified in a NanoDrop spectrophotometer (Thermo Scientific^®^). All RNA was treated with TURBO™ DNase (Invitrogen^®^), according to the manufacturer’s recommendations.

Reverse transcription was performed with 10 U of RNase inhibitor, 40U of MMLV-RT, 1X of MMLV-RT buffer, 200 μM of dNTPs (dGTP, dATP, dTTP and dCTP), 126 random hexamer oligonucleotide holes as primers, 20 μL of DEPC water (Invitrogen^®^) and 1 μL of RNA; all were kept at 37°C for one hour to obtain complementary DNA (cDNA). Subsequently, 3 μL of cDNA was used for amplification in a 25 μL reaction volume comprising: 0.625U of Taq DNA polymerase, 5 mM MgCl_2_, 200 μM of dNTPs and 4 pmoles of each primer (**Table 1**) (Invitrogen^®^). Amplification and electrophoresis were performed as described by Birk et al. (2012).

**Table 1:** *Primers* used to verify the production of transcripts for *ciaB* and *p19* genes by *Campylobacter jejuni* in VT and VBNC.

Genes	Sequence 5’→3’	Amplicon size (pb)	Reference
*ciaB*	ATATTTGCTAGCAGCGAAGAG	157	(Li et al., 2008)
	GATGTCCCACTTGTAAAGGTG		
*p19*	GATGATGGTCCTCACTATGG	206	(Birk et al., 2012)
	CATTTTGGCGTGCCTGTGTA		

The quantitative analysis of gene transcription was performed in Bionumerics 6.6 programmthat allowed a two-dimensional densitometric evaluation compared to the bands obtained with the VT and VBNC strains. The values obtained were used, together with the standards provided by the molecular weight marker, in determining the equation of the straight line with R_2_ > 0.99 to calculate the concentration of transcripts in ng/μL.

### 2.7 Cell culture

We removed the liver from five chicken embryos at 14 days of incubation. Then, to remove the red blood cells, we intensely cut the liver, washed and discarded the supernatant (3 times) and added 0.25% trypsin to promote enzyme breakdown on shaking for 10 minutes. We then removed the supernatant from the decant and added 199 (Gibco™) media plus 20% fetal bovine serum (FBS), which was centrifuged at 900 X g for 10 minutes. The pellet was resuspended in 199 media plus 10% FBS and amphotericin B antibiotic (amphotericin B (2.5 uG/mL), gentamicin (50 uG/mL), streptomycin (100 mcg/mL) and penicillin (100 U/mL). Finally, the medium containing the cells was filtered and homogenised, and we counted the cells in the Newbauer Chamber using an optical microscope (Olympus^©^).

The cell suspensions were seeded on a glass coverslip inside a 12-well plate at 1.43 × 104 cells/well density and cultured at 41.5°C and 5% CO_2_ for 36 hours. After confluence, the cells were challenged with 2.8 log CFU (1.43 × 10^4^ cells per well) of two strains of *C. jejuni* in VT and VBNC forms in triplicate. A negative control group (cells treated with the bacterial diluent (PBS) alone) and a positive control group (cells treated with the viable forms of *C. jejuni* IAL8323) were added.

After a period of 24 hours, the cells were washed three times with PBS and treated with Yo Pro-01 (YP) and propidium iodate (PI) (1:1000 each) (Invitrogen) for 30 minutes at room temperature to stain the cells in the apoptosis and necrosis phase. Then, we washed the cells three times and fixed them with 4% formalin for 10 minutes. After that, the cells were treated with Hoechst (Sigma) to stain the DNA of the cell. We washed the slides and arranged them on a ProLong (Invitrogen) anti-smearing slide. We counted the cells under a fluorescence microscope (EVOS FL Cell Imaging System, Life Technologies Corporation, Carlsbad, California, USA), evaluating four fields per slide. We considered the DAP-labelled cells as the total number of cells. The apoptotic cell index was calculated by the ratio between the number of cells stained only by green and blue colour.

### 2.8 Metabolomic evaluation

The test was performed with one of the *C. jejuni* strains kept in suspension in VT form and VBNC form until freeze-drying. For extraction of metabolites, 1000 µL of spectroscopic grade methanol was added to the lyophilised material and homogenised in a vortex for 5 minutes. The material was centrifuged for 15 minutes at 13,000 g, and the supernatant was transferred to another Eppendorf, which was subjected to vacuum concentrator for 30 minutes. The material was resuspended in 400 µL of methanol and filtered through a 0.22 micrometre filter.

Metabolite analyses were performed using an Agilent 7890B GC System/5977B GC/MSD technologies instrument. Separation was provided by a capillary column (Agilent DB-5HT 30 m x 250 µm x 0.25 µm) with high purity helium at a constant flow rate of 1.2 mL min^-1^. The following GC conditions were used: the initial column temperature was maintained at 60°C for 2 minutes, then increased to 280°C at 5°C min^-1^ and then maintained for 30 minutes. The mass spectrometre was operated in full scan mode from 50 to 550 m/z. The transfer line to the mass spectrometre was heated to 240°C and the quadrupole to 150°C. The injection volume was 6 µL.

MassHunter Qualitative v. 10.0 software was used to process the raw data. A ‘Molecular feature extraction (MFE)’ tool was used for extraction of the mass spectra and conversion to.CEF extension. Agilent Mass Profiler Professional (MPP) software v. B.13.1.1 was used to filter and analyze the extracted molecule compounds. The filters used were minimum absolute abundance = 5,000 counts and all allowable charges. The analysis parameters were retention time tolerance of 0.15 min; mass window 15 ppm + 2 mDa. Molecular compounds considered were those present in 100% of at least one group.

### 2.9 Statistical analysis

The results were subjected to an initial percentage descriptive analysis. For comparative tests, the normality of the data was verified, followed by the application of Fischer’s exact test for comparison of two variables and one-way ANOVA or Kruskal-Wallis when comparing three or more variables.

The percentage of necrotic cells was calculated by the ratio between the number of red and blue stained cells. We analyzed the total number of cells by the average number of cells stained in blue. Thus, we used ANOVA followed by Tukey’s test, comparing each group with the negative control group. In addition, we used chi-square and Fisher’s test followed by the binomial between two proportions for analyses of apoptosis and necrosis, comparing each group with the negative or positive group or the VT and VBNC. We evaluated our data with a 95% confidence level using GraphPad Prism 9.0.

The statistical analyses of the data generated in the metabolomic analysis were performed with log^2^ transformed values. The Mann-Whitney test was applied with p-value < 0.05 and fold change equal to or greater than 2.00. The metabolites were identified using the METLIN 2019 database (included in MPP).

## 3 Results

### 3.1 Acquisition of the VBNC form

The assays to evaluate the viability and acquisition of the VBNC form were conducted under nutritional, thermal and oxidative stress conditions from an average amount of 7.8 ± 0.4 log CFU/mL of the bacteria in its VT form, equivalent to 9.7x10^7^ CFU/mL. The results showed that all strains maintained culturability until day eight and then all were induced to the VBNC state over the time of stress exposure.

**Figure 1:**
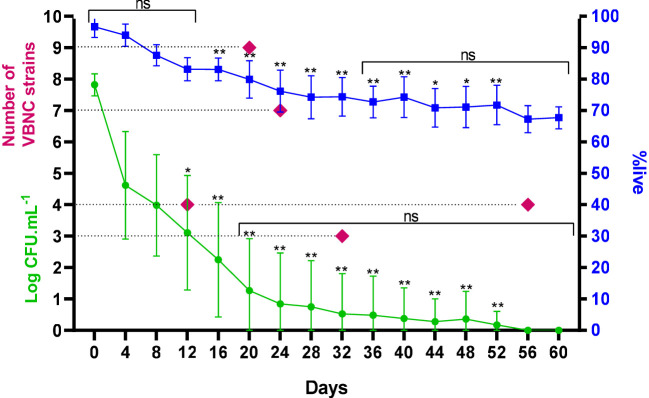
Frequency, count and %live of 27 strains of *C. jejuni* over time under stress until acquisition of the VBNC form. ns, not significant in comparative analysis between days; *p < 0.05; **p < 0.01: in comparative analysis between strains; using a Kruskal-Wallis test. Error bars indicate the standard deviation for the mean counts and percentages obtained.

The average time taken for *C. jejuni* to become VBNC was 26 days. Overall, most strains (16/27 - 59.3%) took 20 and 24 days to acquire the VBNC form. In a discriminated way we had four strains that acquired the VBNC form at 12 days, nine strains at 20 days, seven strains at 24 days, three strains at 32 days and four strains at 56 days. For all strains we observed, the change from the spiral to the coccoid form happened over time, with the concomitant presence of both morphologies in the last eight and four days prior to the complete acquisition of the VBNC form for all strains, at which time the average colony growth was restricted to an average of 0.5 log CFU/mL, equivalent to 3.0 CFU/mL. This shows that the transition is a gradual process, when considering the same strain (**Figure 1**).

Cells were considered VBNC when they were no longer culturable but remained viable in the BacLight assay. The assays in this analysis were performed considering the 27 samples with three results obtained on each day of analysis, with an average variation of 96.6% (with a maximum of 100% and minimum of 87.1%) on day 0 and 67.7% (with a maximum of 71.6% and minimum of 61.4%) on day 60 (**Figure 1**).

The behavior of the strains was similar throughout the first eight days under stress, with the fourth day determining the most significant mean reduction of the culturable form of 3.2 log CFU/mL, in order to maintain the %live without significant changes until day 12. For the other days, this reduction was constant, with an average value of 0.33 log CFU/mL. Starting on day 12, we observed distinct counts of the culturable form among the strains in the same period, which determined the variations in the time of acquisition of the VBNC form. The same was observed for %live from day 16. From day 20 to 60, there was no significant difference in the counts for the strains that maintained their culturability, as well as in viability from day 36 onwards, with an average of 70.8 ± 1.9% (**Figure 1**).

**Figure 2:**
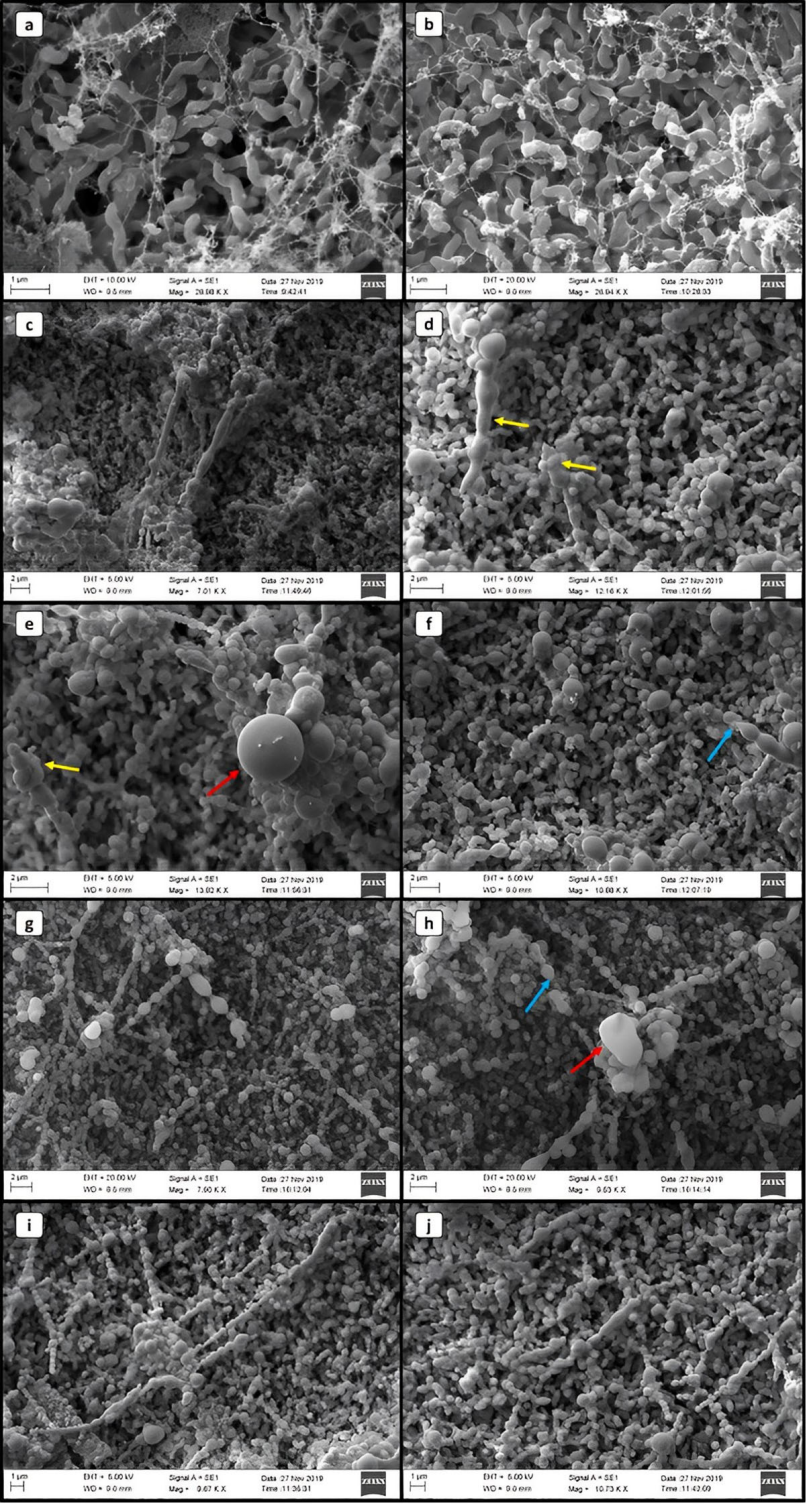
Scanning electron microscopy images of *Campylobacter jejuni* in different life forms. **(A, B)** VT. **(C–J)** CNV. Yellow arrows indicate changes in the cell wall. Red arrows highlight cocci with a diameter of approximately 2 μm. Blue arrows indicate the possible basal structure of the flagellum.

The VT form demonstrated the expected morphological pattern with the helical and spiral structure, typical of the species, with size variations of 0.5–5 µm in length by 0.2–0.8 µm in width, with a flagella at one or both ends (**Figures 2A, B**). The VBNC form showed reduced size and rounded, coccus-like shapes, approximately 0.5 μm in diameter, without the presence of a flagella (**Figures 2E, I, J**).

In the SEM images of *C. jejuni* VBNC, we observed moments that indicate the morphological transition. We identified rod-like structures, up to 1 μm wide and up to 8 μm long (**Figures 2C, D**), with the presence of the basal structure of the flagellum (**Figures 2F, H**), which suggests its loss. At more advanced stages, we observed *C. jejuni* with subdivisions into imperfect coccus-like clusters arranged in a chain, with two to eleven structures in sequence, or individual and of quite variable sizes, reaching up to 2 μm in diameter (**Figures 2F–H**). Fragmentation-like or crust-like changes in the bacterial cell wall structure was also visible at times (**Figures 2D, E**).

**Figure 3:**
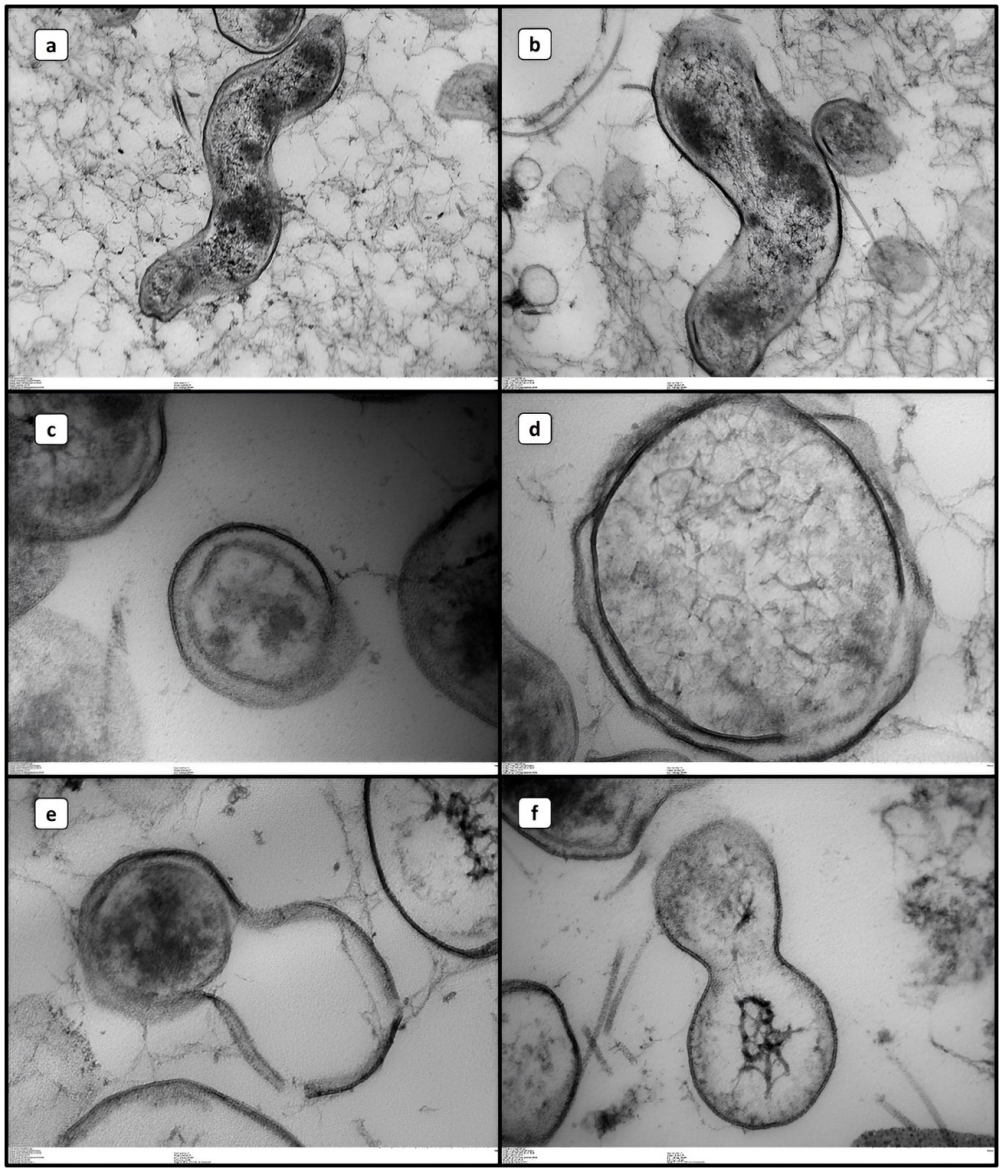
Transmission electron microscopy images of *Campylobacter jejuni* in different life forms. **(A, B)** VT. **(B–F)** VBNC.

In TEM, we detected the typical internal structures of *C. jejuni* VT (**Figures 3A, B**) and VBNC (**Figures 3C–F**), including the capsule, cell wall, ribosomes, genetic material and flagella in some cases. In a comparative manner, we observed that *C. jejuni* VBNC showed a more contrasted and thicker cell wall compared to that observed in the VT form, indicating some protective mechanism of the bacterium (**Figures 3C–E**). In addition, the chromosomal DNA of these cells showed less condensation in general, indicating low cellular activity (**Figures 3D, F**).

### 3.2 Transcriptional analysis

We detected the presence of transcripts for *p19* in all strains in both conditions (VT and VBNC). For the *ciaB* gene, the transcript was only complete for the VT of *C. jejuni* and in 16/27 (59.3%) strains in the VBNC form, indicating inactivation of virulence ability (*ciaB*) in 11/27 (40.7%) and maintenance of adaptive ability under stress (*p19*) in the VBNC form with the presence of transcripts in 100% of the strains. 13/16 (81.2%) strains that produced transcripts for *ciaB* in the VBNC form represent the 13 strains that acquired this stage most rapidly (12 and 20 days), and for 3/16 (18.8%), this acquisition occurred at the last detectable time of our study (56 days) and represent 3/4 strains belonging to this group. Paradoxically, the faster acquisition of loss of cultivation capacity maintained the invasive potential of the strains, as well as strains that remained viable and cultivable for long periods also transcribed *ciaB* when VBNC form.

**Figure 4:**
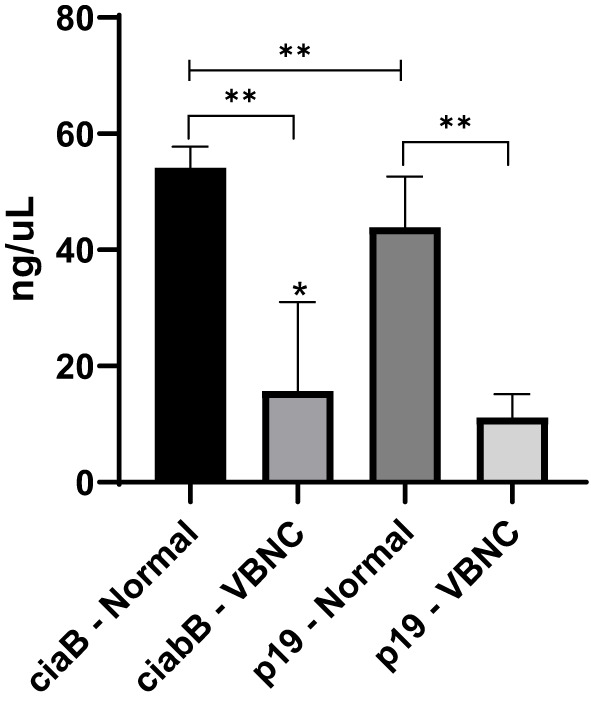
Quantitative analysis of *ciaB* and *p19* transcript regulation in normal and VBNC forms of 27 strains of *C. jejuni*. *p < 0.05 within the same group; **p < 0.01 between groups, using one-way ANOVA. Error bars indicate the standard deviation for the means obtained by eight replicates by strain.

Quantitative analysis of gene transcription revealed the occurrence of significant downregulation for both genes in *C. jejuni* VBNC. For the VT, the *ciaB* transcript was significantly higher (mean 54.2 ng/uL) compared to *p19* (mean 43.9 ng/uL); whereas, for the VBNC stage, the means were 15.7 and 11.2 ng/uL of transcripts for the respective genes (**Figure 4**).

### 3.3 Changes in cell culture

**Figure 5:**
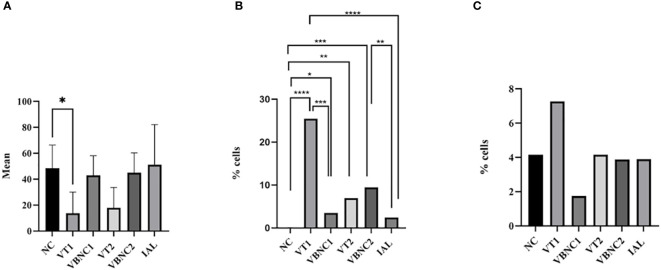
Mean of total cells **(A)** and percentage of cells in apoptosis **(B)** or necrosis **(C)**. We considered the cells marked with DAP as the total number of cells. The apoptotic cell index was calculated by the ratio between the number of cells that were stained only by green and blue. The percentage of necrotic cells was calculated by the ratio between the number of cells stained in red and blue. We used ANOVA followed by the Tukey test, comparing each group with the negative control group. We used the chi-square and Fisher’s tests, followed by binomial between two proportions for apoptosis and necrosis analyses, comparing each group with the negative or positive group or typical and VCN form (< 0.05). NC, negative control. VT1, VT2: *C. jejuni* VT. VBNC1, VBNC2: *C. jejuni* VBNC. *p < 0.05; **p < 0.01; ***p < 0.001 and ****p < 0.0001.

The total number of cells was lower in the VT1 group compared to the negative control group (**Figure 5A**). We found a higher percentage of cells stained with YP in VT1, VBNC1, VT2 and VBCN2, compared to the negative control group (**Figure 5B**). VT1 and VBNC2 showed higher levels of apoptosis than the positive control (IAL) (**Figure 5B**). Apoptosis in VT1 was higher than VBNC1 (**Figure 5B**). However, VT2 and VBNC2 had similar apoptosis rates. We found no difference in the necrosis ratio between groups (**Figure 5C**).

### 3.4 Analysis of bacterial metabolites

**Figure 6:**
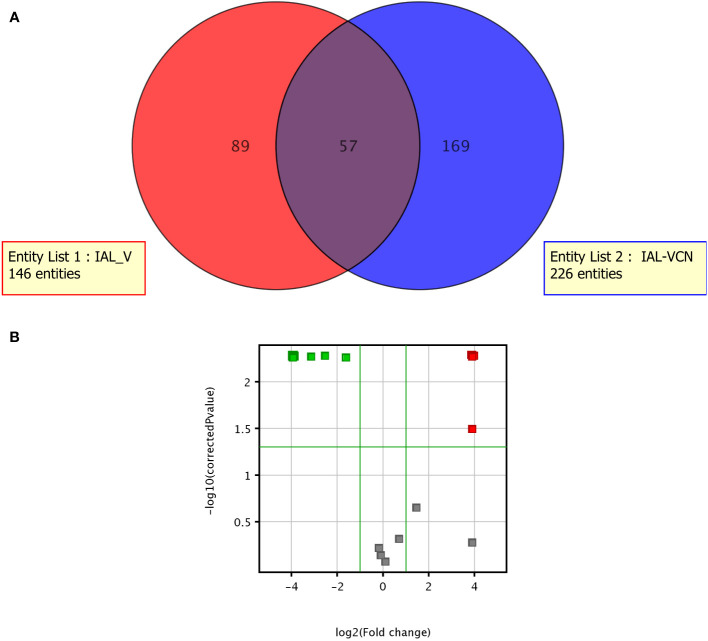
Metabolome of *C. jejuni* VT and the VBNC. **(A)** Venn diagram; **(B)** Volcano Plot demonstrating the most expressed (in red) and least expressed (in green) metabolites in VT compared to VBNC.

**Figure 7:**
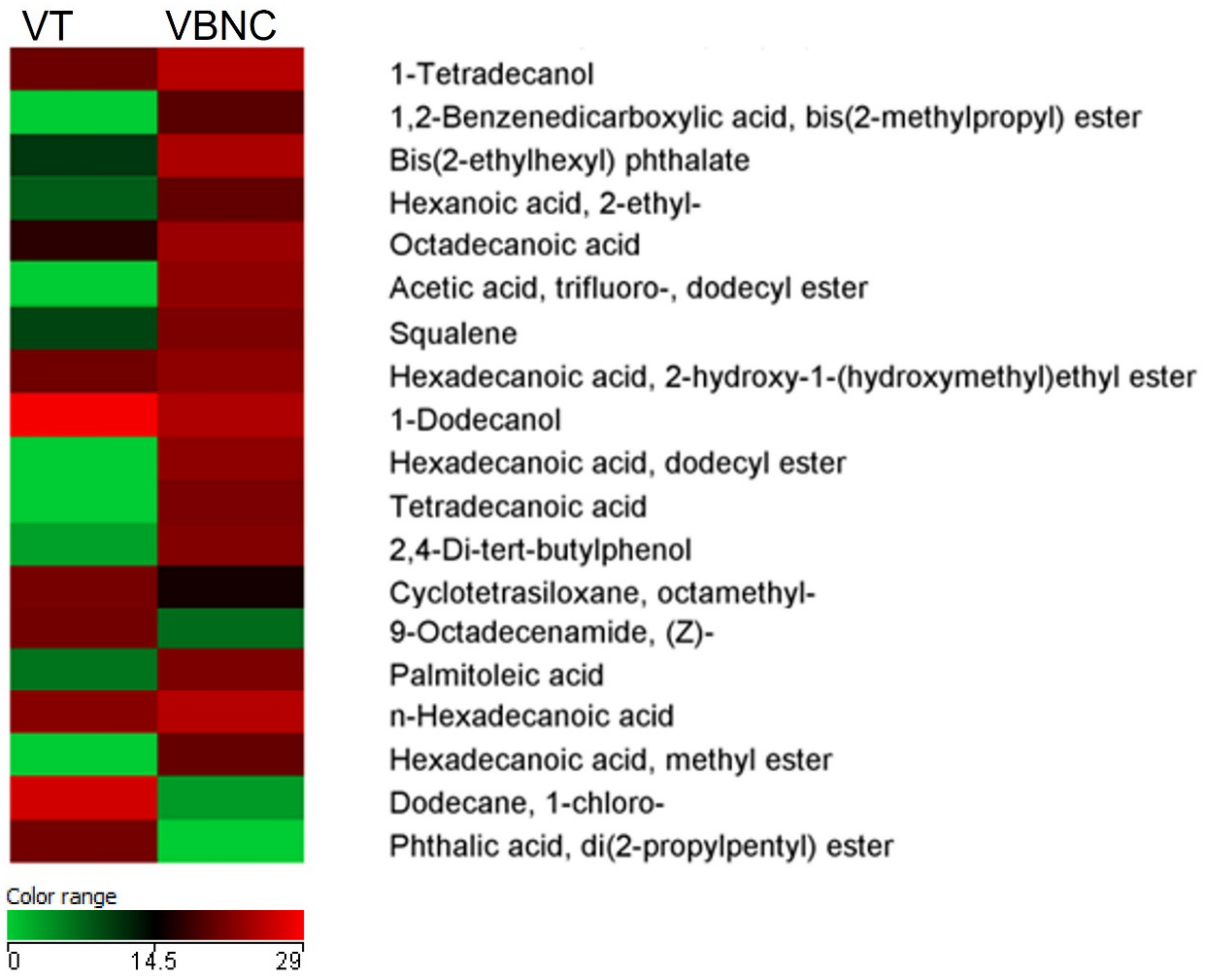
Heatmap of the quantitative variations of the selected metabolites based on the comparison between typical viable (VT) and viable non-culturable (VBNC) *C. jejuni*. Only compounds found in 100% of biological samples within at least one group were considered. Mann-Whitney, p < 0.05. Fold change > 2.00: 27 compounds, of which 19 have relevance to bacterial metabolism.

The metabolome of *C. jejuni* was measurably distinct in the different life forms. A total of 315 metabolites were identified, of which 57 were part of the intersection of compounds common between the two life forms. Of the compounds identified, 89 were shown to be unique to *C. jejuni* VT and 169 were unique to the VBNC form (**Figure 6A**). Twenty-seven metabolites showed significantly distinct expression between the VT and VBNC forms (**Figure 6B**). Eight of these compounds are part of a common set of metabolites released from the vessel and maintenance medium of *C. jejuni* and were therefore excluded from the analysis. We detected that 13 of the 19 metabolites were excreted significantly high in the *C. jejuni* VBNC (**Figure 7**).

Phthalic compounds were identified in both life forms and include high levels of phthalic acid, di(2-propylpentyl) ester in the VT and peaks of phthalate derivatives that include 1,2-benzenedicarboxylic acid bis(2-methylpropyl) ester and bis(2-ethylhexyl) phthalate in the VBNC form.

Metabolites associated with protective mechanisms were found at high levels in *C. jejuni* VBNC and concern squalene, 2,4-di-tert-butylphenol, acetic acid, cyclotetrasiloxane octamethyl, palmitic acid and palmitoleic acid. Similarly, some precursors of volatile organic compounds, belonging to the bacterial volatilome, such as octadecanoid acid, hexanoic acid and tetra decanoic acid, represented an indicative of disruption of the normal metabolism of the cell in the VBNC form.

In the VT form of *C. jejuni*, we can highlight the high levels of dodecane, 9-octadecenamide and di(2-propylpentyl) ester, consistent with the exponential and stationary growth phases representing the metabolism of the bacterium in this life form.

## 4 Discussion

### 4.1 Viability in *C. jejuni*

Obtaining the VBNC form identified in all strains in our study indicates danger to public health because the food may be released as safe, harboring the pathogen that can resume its spiral form when inside the host. According to Reis (2015), bacteria of the genus *Campylobacter* spp. are difficult to grow, and when they are subjected to stress situations, such as food refrigeration or freezing, a morphological transition to the coccoid form occurs, especially in the stationary phase of growth.

This was the first study to determine the mean time to acquisition of the VBNC form in wild strains associated with an analysis of gene transcription, invasion and metabolomics. We found that the VBNC form was identified in a mean time of 26 days within the identified range of 12 to 56 days. A study by Chaisowwong et al. (2012) with a *C. jejuni* strain, isolated from clinical samples from humans in Thailand, determined the need for 38 days for the acquisition of the VBNC form. Our results coupled with those of Chaisowwong et al. (2012) highlight that acquisition of the VBNC form is a strain-dependent trait. VBNC cells are recognised to coexist with both culturable and dead cells in bacterial cultures maintained under standard laboratory conditions (Ayrapetyan et al., 2018). Using the BacLight system at the time when we exclude the culturable cells, no longer detected in traditional cultivation, allows SYTO 9 and PI emissions from bacterial samples to vary depending on the relative concentration of live and dead bacteria in the sample, and this determines the influence on the overall photodegradation rate of the samples. The adjusted dye ratio is considered a simple method that provides the approximate indication of the proportion of live bacteria present in an approximate quantitative of 10^8^ CFU/mL (Ou et al., 2019), which is the reason that determined our evaluation from this quantitative value of bacteria.

Joint oxidative, thermal and nutritional stress represents an effective approach in inducing bacteria to the VBNC state. In view of this, we observed that in general the cultivable cell counts decreased rapidly at the beginning of the stress treatment, especially considering the first four days and continued over time, but the reduction of viable cell counts was insignificant until 12 days of evaluation (**Figure 1**). After treatment under stress for up to 56 days, no cultivable *C. jejuni* cells were detected by the plating assay (< 1 CFU/mL). Stress resistance and progression to the VBNC state is known to vary between different strains of *C. jejuni*, due to the level of stress tolerance (Lv et al., 2020), which appears to be higher in wild-type strains. The ability of these cells to remain viable under temperatures and for such prolonged periods needs further investigation as it raises food safety concerns. The morphology identified in the VBNC form left evident alteration at the level of the bacterial cell wall, cell membrane and capsule. In this life form, several metabolic alterations are identified in *Campylobacter* including the composition of the bacterial external structure that reflects in protection of the microorganism. This general mechanism of tolerance and persistence, with remodeling in size and cell wall thickening, were also found in other microorganisms, such as *Staphylococcus aureus* (Pascoe et al., 2014; Conlon et al., 2016) and *Vibrio parahaemolyticus* (Coutard et al., 2007).

A previous study showed that the alteration in this region is due to poly-P metabolism, which consists of phosphate residues that are linked by high-energy phosphoanhydride bonds that play important roles in bacterial survival and stress tolerance by altering the composition of the bacterial capsule (Gangaiah et al., 2009).The cluster forming pearl necklace-like structures in the VBNC form, with several cocci in sequence, suggests a possible union or even division of the microorganism. Such organization can be explained as a survival strategy in the environment (Abulreesh et al., 2017).

### 4.2 Transcription of virulence and stress adaptation genes

To elucidate the expression potential of virulence and resistance to adverse conditions, we demonstrated the transcription of *ciaB* and *p19* genes, respectively. These genes are related to pathogenicity and adaptive resistance mechanisms of *C. jejuni*, respectively, and encode proteins for these mechanisms, becoming virulence factors of this species (Zheng et al., 2006). According to Chang et al. (2011); Han et al. (2019), and (Rivera-Amill et al., 2001), transcription of the *ciaB* gene is correlated with the invasion of intestinal epithelial cells, and it encodes the *ciaB* protein, linked to the export of flagellin that generates the destruction of microtubules, causing the entry and mobility of the bacterium in the cells of the host organism. The *p19* gene is related to iron transport at the intracellular level during stressful situations because it encodes the iron-dependent periplasmic protein that transports iron. Iron catalyses biochemical reactions necessary for microorganisms, being a cofactor in DNA synthesis and electron transfer (Palyada et al., 2004; Birk et al., 2012). The *p19* protein protects cellular components such as cytoplasmic enzymes, DNA and membrane factors against oxidation (Stintzi and Whitworth., 2003; Monteiro, 2013).

Both genes were selected in the transcription analysis in the VBNC form due to the previous investigation that proved the presence of genes and transcripts in the 27 strains in the VT form, associated with the relevance of both in different functions in *C. jejuni* – *ciaB* (invasion) and *p19* (iron uptake) – and the importance verified in the literature. (Lee et al., 2019; Melo et al., 2019a; Melo et al., 2019b; Askoura et al., 2020; Monteiro et al., 2020; Bravo et al., 2021; Quino et al., 2022).

The transcription of the *ciaB* gene was reduced, considering that 100% of the strains produced transcripts when in the VT and 16/27 (59.3%) in the VBNC form. Nutritional, thermal and oxidative stress induced the decrease of virulence gene transcription. And, in contrast, *p19* transcription was 100% in the VBNC form, maintaining the potential found in the VT form. Therefore, VBNC bacteria regulate and control the transcription of virulence genes that are unnecessary for survival in a stressful environment and prioritise the transcription of genes linked to adaptive processes, such as *p19*. This same behavioural parameter was identified by Moen et al. (2005) when investigating global gene expression patterns of *C. jejuni* grown at 5°C for up to 7 days, finding a negative regulation in the expression of flagellin genes (*flaA* and *flaB*), linked to virulence. In contrast, many genes involved in energy metabolism increased at 5°C, indicating that *C. jejuni* may require higher energy to survive under low temperatures.

### 4.3 Effect of *C. jejuni* VBNC on liver cells

Strain VT1 is probably more pathogenic since the number of cells stained by DAP was lower than the negative control (**Figure 5A**). This result shows that there was cell death and the dead cells probably detached from the surface of the coverslips. Cells marked only with YP are cells in earlier stages of apoptosis, still metabolically active in which DNA fragmentation has not yet occurred in these cells but with compromised plasma membranes (Fujisawa et al., 2014). Although apoptosis is an important mechanism that can regulate immune response (Winoto, 1997) and promote immune tolerance (Griffith et al., 1996), it can also participate in the pathogenic process as a proinflammatory mechanism (Cullen et al., 2013) or an essential strategy for the microorganism to evade the immune system (Mostowy and Cossart, 2012).

Apoptosis is a pathogenic mechanism of *Campylobacter jejuni* (Zhu et al., 1999; Ohara et al., 2004; Siegesmund et al., 2004; Hickey et al., 2005; Butkevych et al., 2020). It can lead to cytotoxicity (Hickey et al., 2005), reduction of the transepithelial resistance, loss of water and electrolytes and increased permeability for macromolecules (Bojarski et al., 2001). The result of apoptosis in our work shows that all strains in typical and VBNC forms have higher potential pathogenicity than the negative or positive control (IAL). Interestingly, the VBNC 1 and VBNC 2 forms showed greater pathogenicity than the positive and negative controls. However, VBNC1 does not follow the pathogenicity of its VT1, while VBNC2 has an apoptotic index identical to VT2 (**Figure 5B**). Our results show that, in VT1, apoptosis contributed to cell pathogenesis since there was a decrease in cells and a high apoptosis index. Although there was no decrease in the number of VBNC1 cells, the VBNC1 forms caused apoptosis in hepatocytes showing that the bacterium was active. We cannot clearly state the effects of VT2 apoptosis, but it is also clear that the VBNC2 form also became metabolically active after contact with hepatocytes.

We found a field strain (VT1) more pathogenic in low doses than a control strain isolated from humans (IAL), which is routinely used in our research. The IAL strain was probably not pathogenic in our study because we used a very low infection dose (2.8 log CFU/well). Furthermore, our results indicate that VBNC forms can be active in the cell culture of hepatocytes. These forms represent a key point in studying the epidemiology of *Campylobacter jejuni* since they are not cultivable.

### 4.4 Metabolomics

Adaptation of bacterial metabolism is crucial for the survival of the microorganism in different environments. Pathogens alter their metabolism to use available resources in the most efficient way and to escape adverse conditions (Behrends et al., 2013).

*Campylobacter jejuni* is considered a microorganism with restricted metabolic capabilities, which justifies the reduced number of compounds found, distinct from what has already been identified for other bacteria, such as *P. aeruginosa*. It is also worth noting that *C. jejuni* exhibits strain-dependent metabolic heterogeneity, with some strains possessing genomic loci that confer different substrate utilization (Mohammed et al., 2004; Line et al., 2010; Stahl et al., 2012). But, comparing closely related strains or strains from the same origin at different developmental stages or life forms seems to be the best experimental approach to investigate these types of changes in bacterial metabolism (Mielko et al., 2019). Of the total 27 metabolites identified as common in both samples, we identified 19 of them, since the rest are part of a common set of metabolites released from the container and maintenance medium of *C. jejuni*. We observed the division between compounds directly related to the bacteria in intense cellular activity and the others related to compounds that justify their production by the need to activate mechanisms of protection, maintenance of cell integrity and blocking of cell metabolism.

Squalene represents a relevant metabolite that relates to acetyl-CoA metabolism and hepanoid biosynthesis. Hepanoids are pentacyclic triterpenoids that are believed to be bacterial surrogates for eukaryotic sterols, acting to stabilize membranes and regulate their fluidity and permeability. Although hopanoids are not essential for growth in several species of bacteria under normal growth conditions, growth defects have been observed under stress conditions and in mutants (van der Donk, 2015). However, the overexpression identified in the VBNC form of *C. jejuni* is indicative of activation of a protective mechanism in this life form, which resulted in visible alterations to the cell wall identified in SEM and TEM (**Figure 2** and **3**). According to Seipke and Loria (2009), some bacterial species modulate their hepanoid content in response to environmental stimuli that allow modification of the composition and in the amount expressed.

Similarly, it is worth mentioning the presence of palmitic acid, the main saturated fatty acid, and palmitoleic acid, the dominant unsaturated fatty acid synthesized by gram-negative bacteria attached to the phospholipids of the cell membrane, as in E. coli (Cronan and Thomas, 2009). The higher expression of these metabolites should probably be associated with a mechanism to reinforce the integrity of the cell membrane in the VBNC form to protect the bacteria.

The presence of fatty acid methyl esters (FAMEs) was identified by the cyclotetrasiloxane octamethyl- metabolite most expressed in the VT form of *C. jejuni* and widely identified in the cell wall of bacteria of the genus *Bacillus* spp. (Sreenivasulu, 2017). It is possible that the reduction of this FAME in *C. jejuni* VBNC is associated with the acidification of the medium or even the intrinsic characteristic of the form. Although we are not aware of other work similar to ours with bacteria of the genus *Campylobacter*, analyses of the genus *Bacillus* and in *P. aeruginosa* show that the FAME profile is positively influenced by the alkaliphilic nature and the bacterial form/lineage. Although not the aim of our work, our results may serve as a basis for further studies using FAMEs as potential life form biomarkers in *Campylobacter jejuni* (Sreenivasulu, 2017).

Volatile amide 9-octadecenamide (oleamide) were identified by Donio et al. (2013a), Donio et al. (2013b) as constituents of a biosurfactant purified from *Bacillus* spp. and now in *C. jejuni* VT form.

The di(2-propylpentyl) ester, a phthalic acid ester (PAE), was overtly identified in *C. jejuni* in the typical form. PAEs have also been detected in other bacterial species such as *H. pylori* and *B. mcbrellneri* (Huang et al., 2021). In parallel, we identified peak phthalate derivatives that include 1,2-benzenedicarboxylic acid bis(2-methylpropyl) ester and Bis(2-ethylhexyl) phthalate in the VBNC form. PAE-linked metabolism is not widely described in bacteria, especially in *Campylobacter*. But, it is possible that there is some mechanism of utilization of these compounds, since the bacterium presents a symporter transporter protein that captures substrates when triggered by the *pmf* gene, with relevant similarity to phthalate family transporters (Sheppard et al., 2013).

The hyper expression of 2,4-di-tert-butylphenol identified in *C. jejuni* VBNC reveals the intense antioxidant activity in this life form. The compound is an alkylbenzene member of the phenol class that carries two tert-butyl substituents at positions 2 and 4. *In vitro* bioassays have demonstrated the ability of this compound to significantly reduce DNA damage produced by the presence of oxygen free radicals, such as those derived from hydrogen peroxide (H_2_O_2_) (Sànchez-Lamar et al., 1999; Sánchez Lamar et al., 2015), which are extremely toxic to *Campylobacter* considering its microaerophilic condition. The compound has already been proven to inhibit H_2_O_2_-induced mutagenesis in bacterial cells (Svobodová et al., 2009), which may contribute to the maintenance of cell viability in *C. jejuni* VBNC.

A wide diversity of metabolites derived from alkane hydrocarbons, associated with other alcoholic or acidic radicals, were identified in both life forms of *C. jejuni*. It is worth noting the relevance of volatile compounds that comprise the bacterial volatilome (Elmassry and Piechulla, 2020). One such compound is dodecane, which represents a volatile organic alkane of recognisable odor produced by microbes. The high concentration of this compound in the viable and VT of *C. jejuni* is consistent with the fact that it is a metabolite produced during the exponential growth phase or in the stationary growth phase, with a key role associated with signaling or virulence of the pathogen. The importance of this metabolite as a marker of diarrheal infection by *C. difficile* and respiratory infection by *A. baumanni* has already been described in the literature (Gao et al., 2016).

In a parallel manner, we identified the higher production of other volatile compound derivatives in the VBNC form. A derivative of octadecanoid acid, 1-Octen-3-ol, identified in our study was determined to be a marker found in patients infected with *C. jejuni* (Garner et al., 2007), as well as hexanoic acid and its derivative, hexadecenoic acid, tetradecane and its derivatives, tetra decanoic acid and tetradecanoyl, which represented metabolites predictive of *C. difficile* infection (Elmassry and Piechulla, 2020). The higher expression of these metabolites in *C. jejuni* VBNC may be due to a disruption of metabolism resulting from environmental stimuli maintaining precursors at doses higher than those identified in the VT form.

High levels of acetic acid have also been identified in blood cultures of *P. aeruginosa* and *E. coli* (Allardyce et al., 2006). In the VBNC form, these high levels are justified by the scarcity of other preferable carbon sources by *C. jejuni* (Stahl et al., 2012), resulting from the nutritional stress condition to which the bacterium was maintained.

Despite reports in the literature regarding the composition of the volatilome of numerous bacterial species, it is still not possible to state the specificity of these metabolites since, besides strain-dependent variation, there is also the involvement of niche, nutrient availability for these pathogens of the storage conditions of the samples and the bacterial growth phase (Nizio et al., 2016; Elmassry and Piechulla, 2020), which may have contributed to the large quantitative variation of the volatile compounds identified in the different life forms of *C. jejuni*.

However, our work opens doors for better phenotypic understanding of VBNC forms of *C. jejuni* and describes possible form biomarkers for this bacterium. The description of such biomarkers is of great epidemiological relevance as it forms the basis for research and innovation studies, especially in the construction of diagnostic methods for the VBNC life form of *C. jejuni* that is not identified by conventional methods.

## 5 Conclusion

*C. jejuni* assumes the VBNC state by thermal, nutritional and oxidative stress after an average of 26 days, with strain-dependent variations. The VBNC form remains potentially virulent, but mainly adapted to stress, as well as regaining its invasive potential in host cells. Image analysis coupled with metabolomics brought explanations about changes in the volatilome and the mechanisms of oxidative protection and cell wall integrity that enhance the maintenance of viability of this life form. These results serve as a public health warning of the potential risk of maintaining this latent form.

## Data availability statement

The original contributions presented in the study are included in the article/supplementary materials. Further inquiries can be directed to the corresponding author.

## Author contributions

DR, BF, MB and RM contributed to conception and design of the study. LS, RB, MG-T, RA, HA-S, LM, PS, JS organized the database and experimental analyses. RM, HA-S, BF performed the statistical analysis. RM, BF, HA-S wrote the first draft of the manuscript. DR, RB, MG-T wrote sections of the manuscript. All authors contributed to manuscript revision, read, and approved the submitted version.

## References

[B1] ABPA (2022) 2022 relatório anual. Available at: https://abpa-br.org/wp-content/uploads/2022/05/Relatorio-Anual-ABPA-2022-1.pdf.

[B2] Abulreesh H. H. Organji S. R. Elbanna K. Haridy Osman G. E. Kareem Almalki M. H. Ahmad I. (2017). Campylobacter in the environment: A major threat to public health. Asian Pacific J. Trop. Dis. 7, 374–384. doi: 10.12980/apjtd.7.2017D6-392

[B3] Allardyce R. A. Langford V. S. Hill A. L. Murdoch D. R. (2006). Detection of volatile metabolites produced by bacterial growth in blood culture media by selected ion flow tube mass spectrometry (SIFT-MS). J. Microbiological Methods 65, 361–365. doi: 10.1016/j.mimet.2005.09.003 16249043

[B4] Askoura M. Youns M. Halim Hegazy W. A. (2020). Investigating the influence of iron on campylobacter jejuni transcriptome in response to acid stress. Microbial Pathogenesis 138, 103777. doi: 10.1016/j.micpath.2019.103777 31600543

[B5] Ayrapetyan M. Williams T. Oliver J. D. (2018). Relationship between the viable but nonculturable state and antibiotic persister cells. J. Bacteriology 200 (20), e00249-18. doi: 10.1128/JB.00249-18 PMC6153661 30082460

[B6] Baffone W. Casaroli A. Citterio B. PierfeliciI L. Campana R. Vittoria E.. (2006). Campylobacter jejuni loss of culturability in aqueous microcosms and ability to resuscitate in a mouse model. Int. J. Food Microbiol. 107, 83–91. doi: 10.1016/j.ijfoodmicro.2005.08.015 16290304

[B7] Behrends V. Ryall B. Zlosnik J. E. A. Speert D. P. Bundy J. G. Williams H. D. (2013). Metabolic adaptations of pseudomonas aeruginosa during cystic fibrosis chronic lung infections. Environ. Microbiol. 15, 398–408. doi: 10.1111/j.1462-2920.2012.02840.x 22882524

[B8] Birk T. Wik M. T. Lametsch R. Knøchel S. (2012). Acid stress response and protein induction in campylobacter jejuni isolates with different acid tolerance. BMC Microbiol. 12, 174. doi: 10.1186/1471-2180-12-174 22889088 PMC3528441

[B9] Bojarski C. Gitter A. H. Bendfeldt K. Mankertz J. Schmitz H. Wagner S.. (2001). Permeability of human HT-29/B6 colonic epithelium as a function of apoptosis. J. Physiol. 535, 541–552. doi: 10.1111/j.1469-7793.2001.00541.x 11533143 PMC2278785

[B10] Bozzola J. J. Russell L. D. (1998). Electron microscopy: Principles and techniques for biologists. 2nd ed (Boston: Jones and Barllet Boston).

[B11] Bravo V. Katz A. Porte L. Weitzel T. Varela C. Gonzalez-Escalona N.. (2021). Genomic analysis of the diversity, antimicrobial resistance and virulence potential of clinical campylobacter jejuni and campylobacter coli strains from Chile. PloS Negl. Trop. Dis. 15, e0009207. doi: 10.1371/journal.pntd.0009207 33606689 PMC7928456

[B12] Bronowski C. James C. E. Winstanley C. (2014). Role of environmental survival in transmission of campylobacter jejuni. FEMS Microbiol. Lett. 356, 8–19. doi: 10.1111/1574-6968.12488 24888326

[B13] Brown H. L. Reuter M. Salt L. J. Cross K. L. Betts R. P. van Vliet A. H. M. (2014). Chicken juice enhances surface attachment and biofilm formation of campylobacter jejuni. Appl. Environ. Microbiol. 80, 7053–7060. doi: 10.1128/AEM.02614-14 25192991 PMC4249011

[B14] Butkevych E. Lobo de Sá F. D. Nattramilarasu P. K. Bücker R. (2020). Contribution of epithelial apoptosis and subepithelial immune responses in campylobacter jejuni-induced barrier disruption. Front. Microbiol. 11. doi: 10.3389/fmicb.2020.00344 PMC7067706 32210941

[B15] CDC (2017). Campylobacter, salmonella levou a doenças bacterianas transmitidas por alimentos em 2016 (Georgia: CDC).

[B16] Chaisowwong W. Kusumoto A. Hashimoto M. Harada T. Maklon K. Kawamoto K. (2012). Physiological characterization of campylobacter jejuni under cold stresses conditions: Its potential for public threat. J. Veterinary Med. Sci. 74, 43–50. doi: 10.1292/jvms.11-0305 21891974

[B17] Chang C. Tasi W. Lai C. Hsu Y. (2011). Association of CiaB with membrane raft-microdomains increases campylobacter jejuni-induced pathogenesis of cells.

[B18] Cisco I. C. Tedesco D. Perdoncini G. Santos S. P. Rodrigues L. B. dos Santos L. R. (2017). Campylobacter jejuni e campylobacter coli EM CARCAÇAS DE FRANGO RESFRIADAS e CONGELADAS. Ciec. Anim. Bras. 18, 1–6. doi: 10.1590/1089-6891v18e-42481

[B19] Conlon B. P. Rowe S. E. Gandt A. B. Nuxoll A. S. Donegan N. P. Zalis E. A.. (2016). Persister formation in staphylococcus aureus is associated with ATP depletion. Nat. Microbiol. 1, 16051. doi: 10.1038/nmicrobiol.2016.51 PMC4932909 27572649

[B20] Coutard F. Crassous P. Droguet M. Gobin E. Colwell R. R. Pommepuy M.. (2007). Recovery in culture of viable but nonculturable vibrio parahaemolyticus: Regrowth or resuscitation? ISME J. 1, 111–120. doi: 10.1038/ismej.2007.1 18043621

[B21] Cronan J. E. Thomas J. (2009), 395–433. doi: 10.1016/S0076-6879(09)04617-5 PMC4095770 19362649

[B22] Cullen S. P. Henry C. M. Kearney C. J. Logue S. E. Feoktistova M. Tynan G. A.. (2013). Fas/CD95-induced chemokines can serve as “Find-me” signals for apoptotic cells. Mol. Cell 49, 1034–1048. doi: 10.1016/j.molcel.2013.01.025 23434371

[B501] Donio M. B. Ronica S. F. Viji V. T. Velmurugan S. Jenifer J. A. Michaelbabu M.. (2013a). Isolation and characterization of halophilic bacillus sp. BS3 able to produce pharmacologically important biosurfactants. Asian Pac. J. Trop. Med. 6, 876–883. doi: 10.1016/S1995-7645(13)60156-X 24083583

[B502] Donio M. B. S. Ronica F. A. Viji V. T. Velmurugan S. Jenifer J. S. C. A. Michaelbabu M.. (2013b). Halomonas sp. BS4, a biosurfactant producing halophilic bacterium isolated from solar salt works in India and their biomedical importance. Springerplus 2, 149. 23667807 10.1186/2193-1801-2-149 PMC3648683

[B23] EFSA (2016). The European union summary report on trends and sources of zoonoses, zoonotic agents and foodborne outbreaks in 2015. ECDC - Eur. Centre Dis. Prev. Control 14, 231. doi: 10.2903/j.efsa.2017.5077

[B24] Elmassry M. M. Piechulla B. (2020). Volatilomes of bacterial infections in humans. Front. Neurosci. 14. doi: 10.3389/fnins.2020.00257 PMC7111428 32269511

[B25] Fujisawa S. Romin Y. Barlas A. Petrovic L. M. Turkekul M. Fan N.. (2014). Evaluation of YO-PRO-1 as an early marker of apoptosis following radiofrequency ablation of colon cancer liver metastases. Cytotechnology 66, 259–273. doi: 10.1007/s10616-013-9565-3 24065619 PMC3918265

[B26] Gangaiah D. Kassem I. I. Liu Z. Rajashekara G. (2009). Importance of polyphosphate kinase 1 for campylobacter jejuni viable-but-Nonculturable cell formation, natural transformation, and antimicrobial resistance. Appl. Environ. Microbiol. 75, 7838–7849. doi: 10.1128/AEM.01603-09 19837830 PMC2794102

[B506] Gao J. Zou Y. Wang Y. Wang F. Lang L. Wang P.. (2016). Breath analysis for noninvasively differentiating acinetobacter baumannii ventilator-associated pneumonia from its respiratory tract colonization of ventilated patients. J. Breath Res. 10, 27102. doi: 10.1088/1752-7155/10/2/027102 27272697

[B27] Garénaux A. Guillou S. Ermel G. Wren B. Federighi M. Ritz M. (2008). Role of the Cj1371 periplasmic protein and the Cj0355c two-component regulator in the campylobacter jejuni NCTC 11168 response to oxidative stress caused by paraquat. Res. Microbiol. 159, 718–726. doi: 10.1016/j.resmic.2008.08.001 18775777

[B505] Garner C. E. Smith S. de Lacy Costello B. White P. Spencer R. Probert C. S. J.. (2007). Volatile organic compounds from feces and their potential for diagnosis of gastrointestinal disease. FASEB J. 21, 1675–1688. doi: 10.1096/fj.06-6927com 17314143

[B28] Griffith T. S. Yu X. Herndon J. M. Green D. R. Ferguson T. A. (1996). CD95-induced apoptosis of lymphocytes in an immune privileged site induces immunological tolerance. Immunity 5, 7–16. doi: 10.1016/S1074-7613(00)80305-2 8758890

[B29] Han X. Guan X. Zeng H. Li J. Huang X. Wen Y.. (2019). Prevalence, antimicrobial resistance profiles and virulence-associated genes of thermophilic campylobacter spp. isolated from ducks in a Chinese slaughterhouse. Food Control 104, 157–166. doi: 10.1016/j.foodcont.2019.04.038

[B30] Hickey T. E. Majam G. Guerry P. (2005). Intracellular survival of campylobacter jejuni in human monocytic cells and induction of apoptotic death by cytholethal distending toxin. Infection Immun. 73, 5194–5197. doi: 10.1128/IAI.73.8.5194-5197.2005 PMC1201269 16041038

[B31] Huang L. Zhu X. Zhou S. Cheng Z. Shi K. Zhang C.. (2021). Phthalic acid esters: Natural sources and biological activities. Toxins 13, 495. doi: 10.3390/toxins13070495 34357967 PMC8310026

[B32] ISO (2017). ISO 10272-1:2017 - microbiology of the food chain – horizontal method for detection and enumeration of campylobacter spp. Microbiology 24. ISO/TC 34/SC 9.

[B33] Lee H. Lee S. Kim S. Ha J. Lee J. Choi Y.. (2019). The risk of aerotolerant campylobacter jejuni strains in poultry meat distribution and storage. Microbial Pathogenesis 134, 103537. doi: 10.1016/j.micpath.2019.05.020 31145980

[B34] Li Y.-P. Ingmer H. Madsen M. Bang D. D. (2008). Cytokine responses in primary chicken embryo intestinal cells infected with campylobacter jejuni strains of human and chicken origin and the expression of bacterial virulence-associated genes. BMC Microbiol. 8, 107. doi: 10.1186/1471-2180-8-107 18588667 PMC2443369

[B504] Line J. E. Hiett K. L. Guard-Bouldin J. Seal B. S. (2010). Differential carbon source utilization by campylobacter jejuni 11168 in response to growth temperature variation. J. Microbiol. Methods 80, 198–202. 20035808 10.1016/j.mimet.2009.12.011

[B35] Lv R. Wang K. Feng J. Heeney D. D. Liu D. Lu X. (2020). Detection and quantification of viable but non-culturable campylobacter jejuni. Front. Microbiol. 10. doi: 10.3389/fmicb.2019.02920 PMC6965164 31998253

[B36] Magajna B. Schraft H. (2015). Evaluation of propidium monoazide and quantitative PCR to quantify viable campylobacter jejuni biofilm and planktonic cells in log phase and in a viable but nonculturable state. J. Food Prot. 78, 1303–1311. doi: 10.4315/0362-028X.JFP-14-583 26197281

[B37] Melo R.T.de Carreon M. M. Monteiro G. P. Mendonça E. P. Peres P. A. B. M. Braz R. F.. (2019a). Maintenance of strains of campylobacter jejuni in laboratories after use of cryoprotectors and pre-treatment of stress. Semina: Ciências Agrárias 40, 3305. doi: 10.5433/1679-0359.2019v40n6Supl2p3305

[B38] Melo R. T. Mendonça E. P. Valadares Júnior E. C. Monteiro G. P. Peres P. A. B. M. Rossi D. A. (2019b). Campylobacter jejuni and campylobacter coli originated from chicken carcasses modulate their transcriptome to translate virulence genes in human cells. Pesquisa Veterinária Bras. 39, 592–599. doi: 10.1590/1678-5150-pvb-6031

[B700] Melo R. T. Grazziotin L. A. Valadares Júnior E. C. Prado R. R. Mendonça E. P. Monteiro G. P. (2019c). Evolution of Campylobacter jejuni of poultry origin in Brazil. Food Microbiol. 82, 489–496. doi: 10.1016/j.fm.2019.03.009 31027810

[B39] Mielko K. A. Jabłoński S. J. Milczewska J. Sands D. Łukaszewicz M. Młynarz P. (2019). Metabolomic studies of pseudomonas aeruginosa. World J. Microbiol. Biotechnol. 35, 178. doi: 10.1007/s11274-019-2739-1 31701321 PMC6838043

[B40] Moen B. Oust A. Langsrud Ø. Dorrell N. Marsden G. L. Hinds J.. (2005). Explorative multifactor approach for investigating global survival mechanisms of campylobacter jejuni under environmental conditions. Appl. Environ. Microbiol. 71, 2086–2094. doi: 10.1128/AEM.71.4.2086-2094.2005 15812042 PMC1082531

[B503] Mohammed K. A. S. Miles R. J. Halablab M. A. (2004). The pattern and kinetics of substrate metabolism of campylobacter jejuni and campylobacter coli. doi: 10.1111/j.1472-765X.2004.01574.x 15287872

[B41] Monteiro G. P. (2013). Viabilidade e expressão de transcritos de virulência em campylobacter jejuni experimentalmente inoculados em queijos minas frescal. Bioscience Journal (Uberlandia). doi: 10.14393/BJ-v36n2a2020-42429

[B42] Monteiro G. P. Melo R.T.de Mendonça E. P. Nalevaiko P. C. Carreon M. M. Buiatte A. B. G.. (2020). Consumption of minas frescal cheese may be a source of human infection by campylobacter jejuni. Bioscience J. 36, 46-52. doi: 10.14393/BJ-v36n2a2020-42429

[B43] Mostowy S. Cossart P. (2012). Bacterial autophagy: Restriction or promotion of bacterial replication? Trends Cell Biol. 22, 283–291. doi: 10.1016/j.tcb.2012.03.006 22555009

[B44] Nizio K. D. Perrault K. A. Troobnikoff A. N. Ueland M. Shoma S. Iredell J. R.. (2016). *In vitro* volatile organic compound profiling using GC×GC-TOFMS to differentiate bacteria associated with lung infections: A proof-of-concept study. J. Breath Res. 10, 26008. doi: 10.1088/1752-7155/10/2/026008 27120170

[B45] Ohara M. Hayashi T. Kusunoki Y. Miyauchi M. Takata T. Sugai M. (2004). Caspase-2 and caspase-7 are involved in cytolethal distending toxin-induced apoptosis in jurkat and MOLT-4 T-cell lines. Infection Immun. 72, 871–879. doi: 10.1128/IAI.72.2.871-879.2004 PMC321583 14742531

[B46] Ou F. McGoverin C. Swift S. Vanholsbeeck F. (2019). Rapid and cost-effective evaluation of bacterial viability using fluorescence spectroscopy. Analytical Bioanalytical Chem. 411, 3653–3663. doi: 10.1007/s00216-019-01848-5 PMC6571086 31049617

[B47] Palyada K. Threadgill D. Stintzi A. (2004). Iron acquisition and regulation in campylobacter jejuni. J. Bacteriology 186, 4714–4729. doi: 10.1128/JB.186.14.4714-4729.2004 PMC438614 15231804

[B48] Pascoe B. Dams L. Wilkinson T. S. Harris L. G. Bodger O. Mack D.. (2014). Dormant cells of staphylococcus aureus are resuscitated by spent culture supernatant. PloS One 9, e85998. doi: 10.1371/journal.pone.0085998 24523858 PMC3921112

[B49] Pascoe B. Méric G. Murray S. Yahara K. Mageiros L. Bowen R.. (2015). Enhanced biofilm formation and multi-host transmission evolve from divergent genetic backgrounds in c ampylobacter jejuni. Environ. Microbiol. 17, 4779–4789. doi: 10.1111/1462-2920.13051 26373338 PMC4862030

[B50] Pinto D. Santos M. A. Chambel L. (2015). Thirty years of viable but nonculturable state research: Unsolved molecular mechanisms. Crit. Rev. Microbiol. 41, 61–76. doi: 10.3109/1040841X.2013.794127 23848175

[B51] Quino W. Caro-Castro J. Hurtado V. Flores-León D. Gonzalez-Escalona N. Gavilan R. G. (2022). Genomic analysis and antimicrobial resistance of campylobacter jejuni and campylobacter coli in Peru. Front. Microbiol. 12. doi: 10.3389/fmicb.2021.802404 PMC8787162 35087501

[B52] Ramamurthy T. Ghosh A. Pazhani G. P. Shinoda S. (2014). Current perspectives on viable but non-culturable (VBNC) pathogenic bacteria. Front. Public Health 2. doi: 10.3389/fpubh.2014.00103 PMC4116801 25133139

[B53] Reis L. P. (2015). Utilização das metodologias imunoenzimática e reação em cadeia da polimerase (PCR) para detecção e caracterização de campylobacter spp. em carcaças de frango, (Brazil: Belo Horizonte). http://hdl.handle.net/1843/SMOC-9TPHBN

[B54] Rivera-Amill V. Kim B. J. Seshu J. Konkel M. E. (2001). Secretion of the virulence-associated campylobacter invasion antigens from campylobacter jejuni requires a stimulatory signal. J. Infect. Dis. 183, 1607–1616. doi: 10.1086/320704 11343209

[B55] Rossi D. A. Dumont C. F. Santos A.C.de S. Vaz M.E.de L. Prado R. R. Monteiro G. P.. (2021). Antibiotic resistance in the alternative lifestyles of campylobacter jejuni. Front. Cell. Infection Microbiol. 11. doi: 10.3389/fcimb.2021.535757 PMC8155616 34055658

[B57] Sánchez Lamar A. Pérezb J. A. Rosb J. Ponsb J. Ferrerc M. Fuentesd J. L.. (2015). Fractionation of an aqueous extract of phyllanthus orbicularis kunth and identification of antioxidant compounds. Rev. Cubana Cienc. Biológicas (RCCB) 4, 56–62.

[B56] Sànchez-Lamar A. Fiore M. Cundari E. Ricordy R. Cozzi R. De Salvia R. (1999). Phyllanthus orbicularis aqueous extract: Cytotoxic, genotoxic, and antimutagenic effects in the CHO cell line. Toxicol. Appl. Pharmacol. 161, 231–239. doi: 10.1006/taap.1999.8814 10620480

[B58] Seipke R. F. Loria R. (2009). Hopanoids are not essential for growth of streptomyces scabies 87-22. J. Bacteriology 191, 5216–5223. doi: 10.1128/JB.00390-09 PMC2725584 19502399

[B59] Sheppard S. K. Dallas J. F. Strachan N. J. C. MacRae M. McCarthy N. D. Wilson D. J.. (2009). Campylobacter genotyping to determine the source of human infection. Clin. Infect. Dis. 48, 1072–1078. doi: 10.1086/597402 19275496 PMC3988352

[B60] Sheppard S. K. Didelot X. Jolley K. A. Darling A. E. Pascoe B. Meric G.. (2013). Progressive genome-wide introgression in agricultural campylobacter coli. Mol. Ecol. 22, 1051–1064. doi: 10.1111/mec.12162 23279096 PMC3749442

[B61] Siegesmund A. M. Konkel M. E. Klena J. D. Mixter P. F. (2004). Campylobacter jejuni infection of differentiated THP-1 macrophages results in interleukin 1β release and caspase-1-independent apoptosis. Microbiology 150, 561–569. doi: 10.1099/mic.0.26466-0 14993305

[B62] Silva J. Leite D. Fernandes M. Mena C. Gibbs P. A. Teixeira P. (2011). Campylobacter spp. as a foodborne pathogen: A review. Front. Microbiol. 2. doi: 10.3389/fmicb.2011.00200 PMC3180643 21991264

[B63] Sreenivasulu B. (2017). Analysis of chemical signatures of alkaliphiles using fatty acid methyl ester analysis. J. Pharm. Bioallied Sci. 9, 106–114. doi: 10.4103/jpbs.JPBS_286_16 28717333 PMC5508411

[B64] Stahl M. Butcher J. Stintzi A. (2012). Nutrient acquisition and metabolism by campylobacter jejuni. Front. Cell. Infection Microbiol. 2. doi: 10.3389/fcimb.2012.00005 PMC3417520 22919597

[B65] Stintzi A. Whitworth. L. (2003). Investigation of the campylobacter jejuni cold-shock response by global transcript profiling. Genome Lett. 2, 18–27. doi: 10.1166/gl.2003.000

[B66] Svobodová A. Zdařilová A. Vostálová J. (2009). Lonicera caerulea and vaccinium myrtillus fruit polyphenols protect HaCaT keratinocytes against UVB-induced phototoxic stress and DNA damage. J. Dermatol. Sci. 56, 196–204. doi: 10.1016/j.jdermsci.2009.08.004 19747801

[B67] ThermoFisher Scientific. LIVE/DEAD® (2004). ThermoFisher scientific . LIVE/DEAD® BacLightTM bacterial viability kits.

[B68] van der Donk W. A. (2015). Bacteria do it differently: An alternative path to squalene. ACS Cent. Sci. 1, 64–65. doi: 10.1021/acscentsci.5b00142 27162951 PMC4827487

[B69] van Vliet A. H. M. Ketley J. M. (2001). Pathogenesis of enteric campylobacter infection. J. Appl. Microbiol. 90, 45S–56S. doi: 10.1046/j.1365-2672.2001.01353.x 11422560

[B70] Winoto A. (1997). Cell death in the regulation of immune responses. Curr. Opin. Immunol. 9, 365–370. doi: 10.1016/S0952-7915(97)80083-0 9203410

[B71] Yahara K. Méric G. Taylor A. J. de Vries S. P. W. Murray S. Pascoe B.. (2017). Genome-wide association of functional traits linked with campylobacter jejuni survival from farm to fork. Environ. Microbiol. 19, 361–380. doi: 10.1111/1462-2920.13628 27883255

[B72] Zheng J. Meng J. Zhao S. SinghI R. Song W. (2006). Adherence to and invasion of human intestinal epithelial cells by campylobacter jejuni and campylobacter coli isolates from retail meat products. J. Food Prot. 69, 768–774. doi: 10.4315/0362-028X-69.4.768 16629018

[B73] Zhu J. Meinersmann R. J. Hiett K. L. Evans D. L. (1999). Apoptotic effect of outer-membrane proteins from campylobacter jejuni on chicken lymphocytes. Curr. Microbiol. 38, 244–249. doi: 10.1007/PL00006795 10069862

